# Treatment patterns and clinical outcomes of chemotherapy treatment in patients with muscle-invasive or metastatic bladder cancer in the Netherlands

**DOI:** 10.1038/s41598-020-72820-y

**Published:** 2020-09-25

**Authors:** Daan J. Reesink, Ewoudt M. W. van de Garde, Bas. J. M. Peters, Paul B. van der Nat, Maartje Los, Simon Horenblas, Harm H. E. van Melick

**Affiliations:** 1 Urology Department, St. Antonius Hospital, Koekoekslaan 1, 3435CM Nieuwegein/Utrecht, The Netherlands; 2 Clinical Pharmacy Department, St. Antonius Hospital, Nieuwegein/Utrecht, The Netherlands; 3 Value Based Healthcare Department, St. Antonius Hospital, Nieuwegein/Utrecht, The Netherlands; 4 Oncology Department, St. Antonius Hospital, Nieuwegein/Utrecht, The Netherlands; 5 Urology Department, The Netherlands Cancer Institute, Amsterdam, The Netherlands

**Keywords:** Cancer, Bladder, Urology, Medical research, Epidemiology, Outcomes research

## Abstract

This retrospective study was performed to evaluate real-world oncological outcomes of patients treated with chemo-based therapy for muscle-invasive or metastatic bladder cancer (MIBC/mBC) and compare results to data from RCTs and other cohorts. Among 1578 patients diagnosed, 470 (30%) had MIBC/mBC. Median overall survival (mOS) for RC alone (47 months), first-line (13 months) and second-line (7 months) chemotherapy, and chemotherapy for recurrent disease (8 months) were similar to literature. Treatment with neoadjuvant and induction chemotherapy (NAIC) was only utilized in 9% of patients, and often in patients with poor disease status, resulting in a lower mOS compared to literature (35 and 20 months, respectively). Patients treated with chemotherapy had many adversities to treatment, with only 50%, 13%, 18% and 7% of patients in NAIC, first-line, salvage after RC, and second-line setting completing the full pre-planned chemotherapy treatment. Real-world data shows NAIC before RC is underutilized. Adversities during chemotherapy treatment are frequent, with many patients requiring dose reduction or early treatment termination, resulting in poor treatment response. Although treatment efficacy between RCTs and real-world patients is quite similar, there are large differences in baseline characteristics and treatment patterns. Possibly, results from retrospective studies on real-world data can deliver missing evidence on efficacy of chemotherapy treatment on older and ‘unfit’ patients.

## Introduction

In general, patients with muscle invasive bladder cancer (MIBC) with or without metastases have a worse prognosis than patients without muscle invasion. MIBC patients treated with radical cystectomy (RC) and extensive pelvic lymph node dissection (PLND) have a median overall survival (mOS) of 46 months, which can be prolonged to 77 months with neoadjuvant chemotherapy (NAC)^1^. Similar results are shown in the ICOT-trial^2^. Although significant survival benefit of these two studies, a meta-analysis on NAC before RC only shows a 5-years survival benefit of 5%-10%, compared to RC alone^1,3^. Patients with advanced and/or metastatic bladder cancer (mBC) have a mOS of 13–16 months, despite having a response rate of 40–60% in the first-line (1L) setting with cisplatin-based chemotherapy^4,5^. Patients receiving second-line (2L) single agents after failure of cisplatin based 1L treatment, have an even worse prognosis with a mOS of 6–7 months, questioning whether there is any benefit at all for OS or quality of life (QoL)^6–8^.

Clinical trials are characterized by having strict in- and exclusion criteria^9^. For example, in the ABC Collaboration analysis on which guidelines rely, only 4% of patients had a Performance Status (PS) of 2–3, and 3% had a renal function (GFR) < 60 mL/min^3^. In contrast to the clinical trial population, it is estimated that only 36% of patients presenting with advanced UC are treated with cisplatin^10,11^, and it is estimated that 40–59% of UCC patients are not eligible for standard cisplatin-based chemotherapy^12,13^. Not only are elderly patients considered more ineligible for cisplatin-based chemotherapy, they are also heavily underrepresented in clinical trials^14–16^. Survival benefits of chemotherapy treatment of these elder and/or ‘unfit’ patients are largely unknown.

Retrospective cohort studies show a wide variety of results on overall survival. A large retrospective cohort study from the US showed a mOS after RC of 48 months, but does not differentiate between upfront RC and neoadjuvant or induction chemotherapy (NAIC) before RC^17^. Niegisch et al. performed a real-world data study in Germany on survival outcomes after 1L and 2L treatment, and found a mOS of 16.1 and 9.2 months, respectively.

In the Netherlands, about 7000 patients are diagnosed with BCa each year^18^. Approximately 25% have MIBC and/or mBC^17^. There are only a few retrospective studies from the Netherlands reporting on survival in the general population^19,20^, and all these studies originate from The Netherlands Cancer Institute (NCI) which is a specialised tertiary care cancer centre, and may not be representative for the general clinical practice. Therefore, the question whether outcomes from treatment options for bladder cancer (BCa) in the general population (so called real-world setting) are similar compared to results from controlled clinical trials, remains unanswered. The primary objective of this retrospective, observational cohort analysis was to describe treatment patterns and outcomes of patients with MIBC or mBC and a chemo-based therapy within a regional referral network for treatment of BCa in the Netherlands.

## Methods

### Study design

A non-interventional, retrospective study was conducted. The study has been approved by the local research ethics committee of the St. Antonius Hospital Utrecht/Nieuwegein (W17.087).

The St. Antonius Hospital is a large 1100 beds teaching hospital within a regional network; it leads multidisciplinary consultation and functions as a referral center for radical cystectomies. Within this network there are yearly almost 200 diagnoses of BCa.

### Patient population

Eligible patients were ≥ 18 years, diagnosed with MIBC or mBC, defined as ≥ cT2N0M0, between 2008 and 2016. Patients were categorized by treatment setting, i.e. RC alone, NAIC + RC, radiotherapy (RTx), and best supportive care (BSC) for MIBC. For mBC, patients were categorized in 1L chemotherapy, salvage chemotherapy after recurrence after RC, 2L chemotherapy and BSC. All MIBC and mBC patients diagnosed in our regional network are discussed in a weekly multidisciplinary setting. Patients with cT2-4aN0-3M0 disease are found eligible for RC with or without NAIC. Patients indicated for (curative or palliative) RTx, are referred to a regional radiotherapeutics center. Patients found eligible for chemotherapy are referred to the oncology department for chemotherapy treatment.

The treatment used in our institute is gemcitabine/cisplatin (GEM-CIS) (gemcitabine 1000–1250 mg/m^2^ and cisplatin 70 mg/m^2^ on day 1 in 4 h, and gemcitabine 1000 mg/m^2^ on day 8 in 30 min in a 21-day cycle. When patients are considered unfit for cisplatin, they received a combination of gemcitabine/carboplatin (GEM-CARBO). Patients received gemcitabine 1000–1250 mg/m^2^, and area under the curve (AUC) 5 carboplatin on day 1, both in 30 min, and gemcitabine same dose on day 8, in a 21-day cycle. Cancer of the upper urinary tract was excluded from analysis. Starting from the end of 2017, patients of our hospital received also immunotherapy. These patients were excluded for this analysis.

### Patient selection and data collection

Patients were identified with help of the Netherlands Cancer Registry (NCR). All patients newly diagnosed with bladder cancer in the St. Antonius Hospital or affiliated hospitals (Zuwe Hofpoort Hospital Woerden, Beatrix Hospital Gorinchem and Diakonessenhuis Utrecht) during the study years were extracted from the database. Subsequently, the hospital’s pharmacy administration system, which contains amongst others, drug name, dosage, date of administration, and administration route, was used to identify chemotherapy treatment. Patients who underwent RC were identified from the St. Antonius Hospital’s prospective cystectomy database. The remaining patients from the NCR database with stage ≥ cT2N0M0 that could not be matched with either database were separately evaluated to find treatment received [including best supportive care (BSC)].

### Patient characteristics and outcome parameters.

The NCR database contained date of birth, vitality status, date of death, morphology, and tumour cTNM stage. By manual chart review, the following variables were extracted: treatment received, date of cystectomy, pTNMR stage after cystectomy, date start chemotherapy, kidney function at start chemotherapy, chemotherapy cycles planned, chemotherapy cycles received, complications during chemotherapy (within 30 days), response to chemotherapy by imaging using the Response Evaluation Criteria in Solid Tumors (RECIST) criteria^21^, recurrence of disease, date of recurrence, treatment for recurrent disease. cTNM stage was checked for authenticity.

Topography and morphology were classified according to the International Classification of Diseases of Oncology (ICD-O) and tumour stage according to the TNM classification system^22^.

Progression-free survival (PFS) was calculated as time difference (in months) between start of treatment (cystectomy, radiotherapy, chemotherapy) and first radiological evidence of progression. OS was calculated as the time difference (in months) between start treatment and date of death (vitality status on 31-12-2018). For BSC, date of diagnosis was used.

Clinical complete response (cCR) was defined as the absence of tumour on cross-sectional imaging. Partial response (cPR), stable disease (cSD), and progressive disease (cPD) were defined as a decreased, similar or increase size of the tumour compared to pre-systemic therapy cross-sectional imaging, respectively^21^. Pathological response on systemic therapy was defined according to a recent publication of Zargar et al., where pCD is downstaging to a non-muscle invasive disease without LN metastases (≤ ypT1N0)^23^.

Early termination of systemic therapy was defined as not receiving the pre-planned amount of cycles. Dose reduction was defined as a dose < 80% of the initial dose, or skipping of a day gemcitabine. Dose delay was defined as delay of the next treatment cycle of > 7 days. A treatment switch was defined as a switch from cisplatin to carboplatin. Full-cycle completion was defined as the absence of early termination, dose reduction or treatment switch. Response to chemotherapy was defined as response at the end of systemic treatment. Toxicity was graded according to the National Cancer Institute Common Terminology Criteria for Adverse Events (CTCAE), version 4.0. Re-admittances and ER-visits were calculated when events occurred during or in < 30 days after ending treatment.

### Statistical analyses

Descriptive statistics were composed for the total sample, and for each treatment setting, including medians and interquartile range (IQR) or 95% confidence intervals [95% CI]. The Kaplan–Meier method was used to determine and compare survival. The reverse Kaplan–Meier method was used to determine median follow-up. Statistical analyses were performed with SPSS statistical software (version 24.0, SPSS Inc. Chicago, IL, United States of America). All reported *P*-values were two-sided and considered statistically significant at < 0.05.

### Ethical approval

All procedures performed in studies involving human participants were in accordance with the ethical standards of the institutional and national research committee and with the 1964 Helsinki declaration and its later amendments or comparable ethical standards.

### Informed consent

Due to the retrospective nature of the study, a waiver from informed consent was obtained.

## Results

In total, 1641 patients diagnosed with BCa were identified from the NCR database. Sixty-three patients (3.8%) had a morphology other than UCC and were excluded from further analysis. Of the remaining 1578 patients, MIBC and mBC was diagnosed in 470 patients (30%). Forty-two patient with missing information on treatment, and ten patients receiving palliative RC were excluded from further analyses.

**Figure 1:**
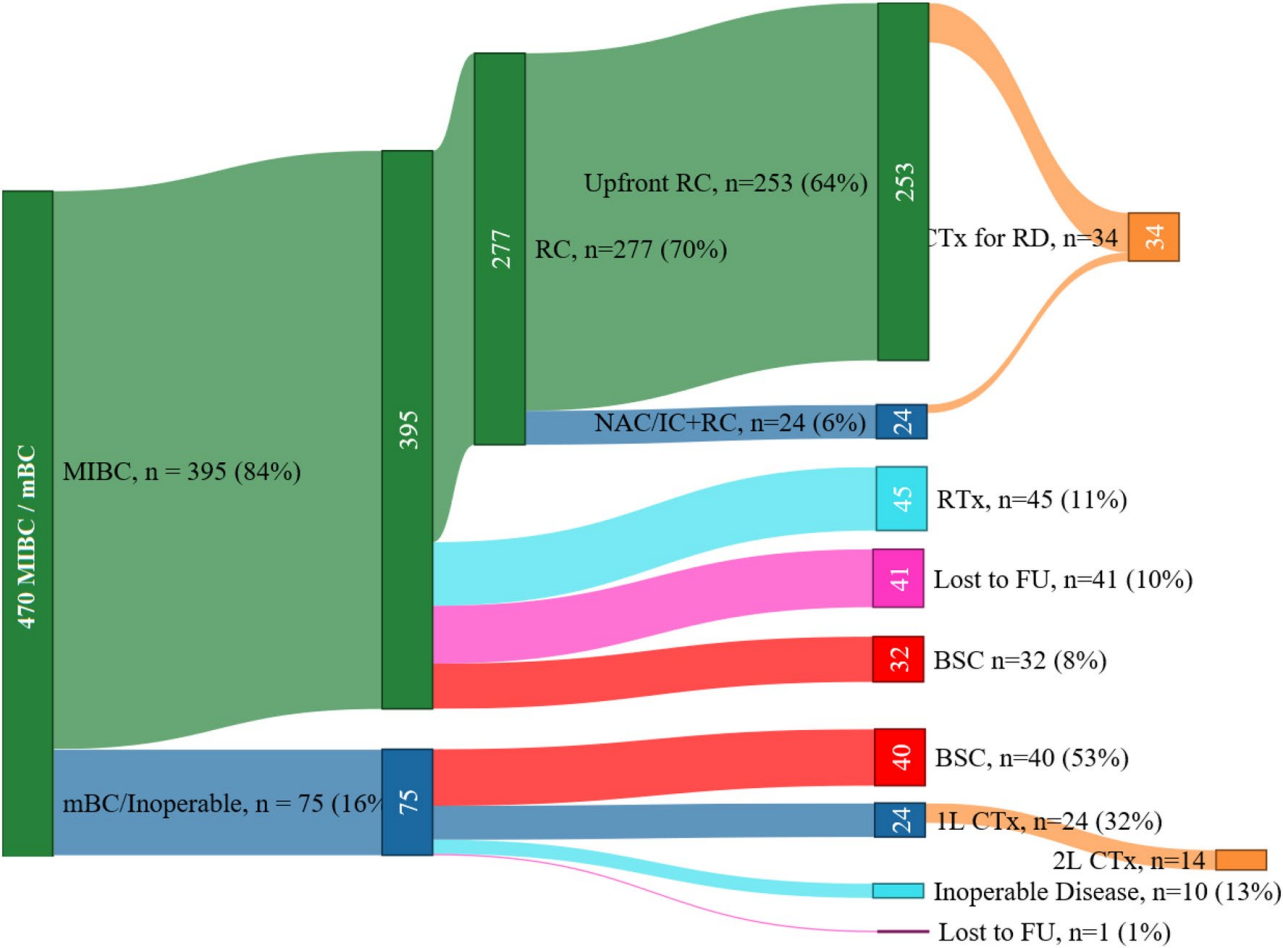
Treatment modalities after primary diagnoses. Sankey Diagram of treatments received. *MIBC* muscle-invasive bladder cancer, *mBC* metastatic bladder cancer, *CTx* chemotherapy, *NAIC* neoadjuvant/induction chemotherapy, *RC* radical cystectomy, *RD* recurrent disease, *RTx* (palliative) radiotherapy, *1L* first-line chemotherapy, *2L* second-line chemotherapy, *FU* follow-up.

Characterisation according to treatment is shown in Fig. 1. Patient characteristics are presented in Table 1. Chemotherapy treatment was planned in NAIC before RC, recurrence after RC, 1L and 2L settings on 96 occasions. Some patients received chemotherapy in more than one setting.

**Table 1:** Patient characteristics.

		Total	Muscle invasive or locally advanced disease				Metastatic or inoperable disease	
			NAIC + RC	RC Alone	Palliative RTx	BSC	1L Chemotherapy	BSC
Total patients		418	24	253	45	32	24	40
Age, median years (IQR)		70.9 (13.9)	62.8 (13.5)	68.2 (12.3)	80.4 (10.7)	81.0 (12.4)	65.4 (7.7)	77.7 (15.2)
**Age, No (%)**								
< 50 years		14 (3.3)	2 (8.3)	11 (4.3)	0 (0.0)	0 (0.0)	0 (0.0)	1 (2.5)
50–60 years		56 (13.4)	7 (29.2)	38 (15.0)	1 (2.2)	2 (6.3)	4 (16.7)	4 (10.0)
60–70 years		126 (30.1)	9 (37.5)	90 (35.6)	3 (6.7)	4 (12.5)	14 (58.3)	6 (15.0)
70–80 years		156 (37.3)	6 (25.0)	106 (41.9)	16 (35.6)	7 (21.9)	5 (20.8)	16 (40.0)
> 80 years		66 (15.8)	0 (0.0)	8 (3.2)	25 (55.6)	19 (59.4)	1 (4.2)	13 (32.5)
**Sex, No. (%)**								
Male		309 (73.9)	18 (75.0)	185 (73.1)	35 (77.8)	21 (65.6)	21 (87.5)	29 (72.5)
**eGFR, No. (%)**								
Unknown		269 (64.4)	0 (0.0)	253 (100.0)	3 (6.7)	3 (9.4)	2 (8.3)	8 (20.0)
≤ 30 mL/min		16 (3.8)	0 (0.0)	0 (0.0)	4 (8.9)	5 (15.6)	0 (0.0)	7 (17.5)
30–60 mL/min		42 (10.0)	3 (12.5)	0 (0.0)	9 (20.0)	12 (37.5)	6 (25.0)	12 (30.0)
≥ 60 mL/min		91 (21.8)	21 (87.5)	0 (0.0)	29 (64.4)	12 (37.5)	16 (66.7)	13 (32.5)
**Clinical TNM-stage, No. (%)**								
T2-4aN0M0		300 (71.8)	6 (25.0)	227 (89.7)	43 (95.6)	24 (75.0)	0 (0.0)	0 (0.0)
T2-4aN+M0		60 (14.4)	16 (66.7)	26 (10.3)	2 (4.4)	8 (25.0)	8 (33.3)	0 (0.0)
T4b or M+		58 (13.9)	2 (8.3)	0 (0.0)	0 (0.0)	0 (0.0)	16 (66.7)	40 (100.0)
**Clinical nodus (N)-stage, No. (%)**								
Nx		37 (8.9)	0 (0.0)	0 (0.0)	12 (26.7)	10 (31.3)	0 (0.0)	15 (37.5)
N0		289 (69.1)	8 (33.3)	227 (89.7)	31 (68.9)	14 (43.8)	1 (4.2)	8 (20.0)
N1		37 (8.9)	1 (4.2)	23 (9.1)	1 (2.2)	3 (9.4)	3 (12.5)	6 (15.0)
N2		45 (10.8)	11 (45.8)	3 (1.2)	1 (2.2)	5 (15.6)	14 (58.3)	11 (27.5)
N3		10 (2.4)	4 (16.7)	0 (0.0)	0 (0.0)	0 (0.0)	6 (25.0)	0 (0.0)
**Clinical metastatic (M)-stage, No. (%)**								
Mx		10 (2.4)	0 (0.0)	0 (0.0)	6 (13.3)	4 (12.5)	0 (0.0)	0 (0.0)
M0		355 (84.9)	24 (100.0)	253 (100.0)	39 (86.7)	28 (87.5)	8 (33.3)	3 (7.5)
M1		53 (12.7)	0 (0.0)	0 (0.0)	0 (0.0)	0 (0.0)	16 (66.7)	37 (92.5)
Follow-up, median months [95% CI]		57.4 [49.4–65.4]	60.2 [29.5–90.9]	60.4 [52.0–68.7]	51.3 [29.2–73.4]	34.3 N.D.*	33.6 N.D.*	9.3 N.D.*
Overall Survival (OS), median months [95% CI]		21.3 [1.7–18.0]	19.5 [0.0–49.1]	47.4 [28.9–65.8]	19.5 [28.9–65.8]	3.8 [3.2–4.5]	12.6 [8.8–16.4]	2.0 [0.9–3.2]

*NAIC* neoadjuvant/induction chemotherapy, *RC* radical cystectomy, *RTx* radiotherapy, *BSC* best supportive care, *1L* first line, *IQR* interquartile range, *95% CI* 95% confidence interval, *eGFR* estimated Glomerular filtration rate.

*95% CI could not be determined.

In 24/277 (8.6%) of patients, NAIC was administered before RC. In patients undergoing RC, mean number of identified number of lymph nodes was 12.6 (SD 6). Of 24 patients starting NAIC before planned RC, four (17%) had progression of disease during treatment, and did not undergo RC. Six patients (25%) started in NAC setting (N0), eighteen patients (75%) in induction (IC) setting (N +). After NAIC + RC, six patients (25%) had ≤ ypT1N0M0. However, three of these patients eventually still had lymph nodal recurrent disease. At study end, three patients with ≤ ypT1N0M0 and two patients with residual muscle-invasive disease after NAIC + RC remained in complete remission at study end (21%).

Chemotherapy characteristics, and treatment outcomes are described in Table 2. For patients receiving chemotherapy, median age (IQR) was 65.6 years (10.1), with 26/96 patients (27%) > 70 years of age. Twenty-four patients (25%) had a renal clearance of < 60 mL/min. In total, 53% of patients were treated with cisplatin-based chemotherapy.

**Table 2:** Chemotherapy outcome results, stratified for treatment setting.

	Total	NAIC + RC	1L chemotherapy	Recurrent disease after RC	2L chemotherapy
Total patients	96	24	24	34	14
**eGFR, No. (%)**					
Unknown	2 (2.1)	0 (0.0)	2 (8.3)	0 (0.0)	0 (0.0)
≤ 30 mL/min	0 (0.0)	0 (0.0)	0 (0.0)	0 (0.0)	0 (0.0)
30–60 mL/min	24 (25.0)	3 (12.5)	6 (25.0)	11 (32.4)	4 (28.6)
≥ 60 mL/min	66 (68.8)	21 (87.5)	16 (66.7)	21 (61.8)	8 (57.1)
**Type of chemotherapy, No. (%)**					
Gemcitabine/Cisplatin	51 (53.1)	20 (83.3)	11 (45.8)	16 (47.1)	4 (28.6)
Gemcitabine/Carboplatin	36 (37.5)	4 (16.7)	13 (54.2)	16 (47.1)	3 (21.4)
Docetaxel	9 (9.4)	0 (0.0)	0 (0.0)	2 (5.9)	7 (50.0)
**Treatment adjustments, No. (%)**					
Dose reduction	47 (49.0)	6 (25.0)	17 (70.8)	20 (58.8)	4 (28.6)
Early termination	35 (36.5)	5 (20.8)	10 (41.7)	13 (38.2)	7 (50.0)
Carboplatin switch	9 (9.4)	3 (12.5)	2 (8.3)	2 (5.9)	2 (14.3)
Full treatment received	22 (22.9)	12 (50.0)	3 (12.5)	6 (17.6)	1 (7.1)
**Best response, No. (%)**					
cPD (clinical progressive disease)	34 (38.2)	6 (25.0)	6 (27.3)	13 (41.9)	9 (75.0)
cSD (clinical stable disease)	8 (9.0)	2 (8.3)	1 (4.5)	5 (16.1)	0 (0.0)
cPR (clinical partial response)	39 (43.8)	15 (62.5)	11 (50.0)	10 (32.3)	3 (25.0)
cCR (clinical complete response)	8 (9.0)	1 (4.2)	4 (18.2)	3 (9.7)	0 (0.0)
Unknown	7	0	2	3	2
CTCAE complication*, No. (%)					
Grade 3	49 (54.4)	8 (36.4)	11 (45.8)	21 (70.0)	9 (64.3)
Grade 4	8 (8.9)	3 (13.6)	3 (12.5)	2 (6.7)	0 (0.0)
Grade 5	4 (4.4)	1 (4.5)	2 (8.3)	0 (0.0)	1 (7.1)
Unknown	6	2	0	4	0
Need for blood transfusion, No. (%)	34 (35.4)	2 (8.3)	9 (37.5)	16 (47.1)	7 (50.0)
Median follow-up [95% CI]	58.9 [33.1–84.8]	58.9 [27.0–90.9]	32.9 N.D.**	50.9 [20.2–81.7]	N.D.**
Median overall survival (OS) [95% CI]	10.2 [7.2–13.2]	19.5 [0.0–49.1]	12.6 [8.8–16.4]	7.5 [0.0–15.6]	6.6 [4.7–8.4]
Median progression-free survival (PFS) [95% CI]	5.6 [4.1–7.1]	9.5 [0.7–18.3]	6.1 [0.0–14.1]	3.5 N.D.**	0.0 N.D.**

*NAIC* Neoadjuvant/induction chemotherapy, *RC* radical cystectomy, *1L* first-line, *2L* second-line, *CTCAE* common terminology criteria for adverse events, *95% CI* 95% confidence interval.

*Maximum grade CTCAE complication that patients suffered.

**Median follow-up and 95% CI could not be determined.

**Figure 2:**
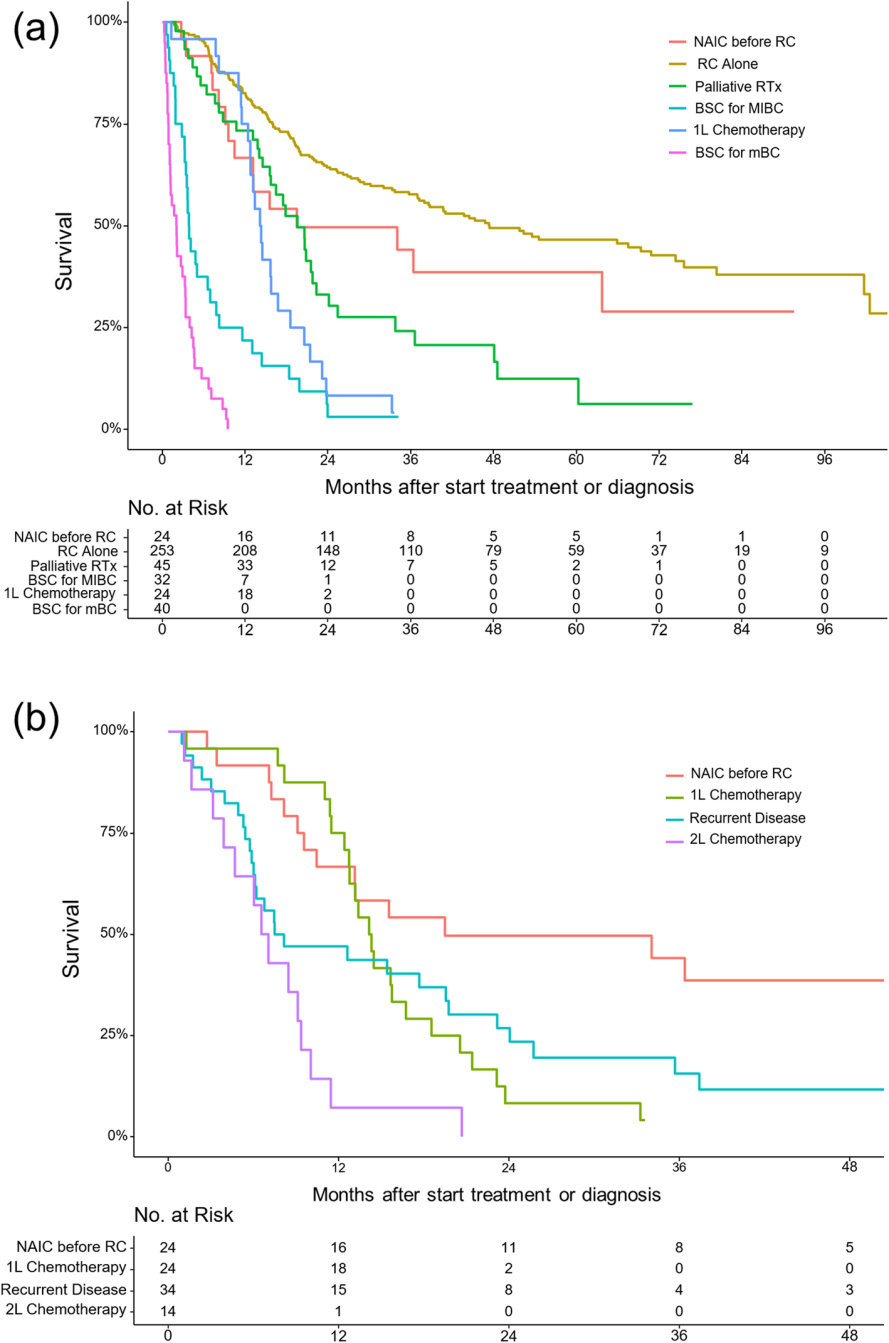
Kaplan–Meier curves, showing overall survival for patients with muscle invasive or metastatic bladder cancer (**a**) stratified for first treatment received, and (**b**) stratified for various chemotherapy treatment settings. *NAIC* neoadjuvant/induction chemotherapy, *1L* first-line chemotherapy, *2L* second-line chemotherapy.

Of all patients treated with GEM-CIS, 14/51 patients (27%) had early termination of treatment, of which 6 patients (43%) due to progression during chemotherapy and 8 patients (57%) due to toxicity. In GEM-CARBO patients, 14/36 patients (39%) had early termination, with also 6 patients (43%) due to progression during chemotherapy and 8 patients (57%) due to toxicity. The overall tumour response rate (cCR + cPR) for all settings combined was 53%. The overall tumour response rate was 67% after NAIC, 68% after 1L chemotherapy, 42% after recurrence after cystectomy and 25% after 2L chemotherapy. Overall survival for each treatment modality is described in Table 2, and are shown in Fig. 2a, b. Supplementary Fig. 3, shows OS differences for GEM-CIS, GEM-CARBO, and Docetaxel if applicable.

In 1L setting, seven patients treated with GEM-CIS (64%) had dose reduction versus ten patients treated with GEM-CARBO (77%). Five patients in both cisplatin-treated group (45%) and carboplatin-treated group (38%) had early termination of treatment cycles. Only one patient had complete response after 1L treatment and was alive at study end with complete remission.

In the chemotherapy for recurrence setting, early termination occurred in four patients (25%) receiving GEM-CIS, compared to eight receiving GEM-CARBO (50%). Dose reduction occurred in eleven GEM-CIS patients (69%), versus nine GEM-CARBO patients (56%). At study end, four patients were alive with stable disease, and two patients with complete remission.

Only one patient in the 2L was able to fully complete the pre-planned chemotherapy treatment. Seven patients were planned for docetaxel treatment, of which one died before start, three patients had progression after one cycle and discontinued treatment. All patients had progression under chemo, with one patient able to receive seven cycles before discontinuing treatment.

A summary of the most frequent complications for each patient is presented in Table 2 and all complications that occurred are summarized in Table 3. Of 96 patients treated with chemotherapy, only 13 (14%) had no complications at all, and 61 (67%) patients had a grade ≥ 3 complication. Different classifications grade ≥ 3 complications occurred 88 times. Admittance for anaemia and blood transfusion was the most occurring complication in 34 patients (35%). In total, transfusion was required 76 times. Fever, requiring hospital admittance, could not be retrospectively discriminated between grade 3 or 4, and was therefore categorized as grade 3. Grade 5 complications occurred in two carboplatin treated patients.

**Table 3:** Frequency of complications.

	Total*	GEM-CIS	GEM-CARBO	Docetaxel
Total patients, No. (%)	96	51	36	9
**Total grade 3 complications, No. (%)**	88	45	37	6
Anemia (transfusion required)	34	16	17	1
Fever, severe (hospitalization)	25	16	7	2
Thromboembolic Event	9	7	2	0
Other	20	6	11	3
**Total grade 4 complications, No. (%)**	11	6	4	1
Fever, life threatening	1	1	0	0
Gastro-intestinal bleeding	5	2	2	1
Thromboembolic Event	2	2	0	0
Myocardial Infarction	1	1	0	0
Other	2	0	2	0
**Grade 5 complications, No. (%)**	4	0	4	0
Sepsis	3	0	3	0
Myocardial Infarction	1	0	1	0

*CTCAE* common terminology criteria for adverse events,* GEM-CIS* Gemcitabine/Cisplatin,* GEM-CARBO* Gemcitabine/Carboplatin.

*Total is more than 100% due to all grade 3–5 complications being calculated, whereas some patients had multiple complications.

In 1L setting, CTCAE grade ≥ 3 complication during treatment occurred in six cisplatin-treated patients (55%), in comparison to ten patients (76%) of carboplatin-treated patients, including two grade 5 complications.

When treated for recurrence after cystectomy, respectively eleven (69%) versus twelve (75%) of the cisplatin- and carboplatin-treated patients had CTCAE grade ≥ 3 complication during treatment.

For 2L chemotherapy, all patients had CTCAE Grade ≥ 3 complications, one patients died of complications from an acute duodenitis. All but one required blood transfusion during treatment.

## Discussion

This study was performed to evaluate oncological outcomes of patients treated with chemotherapy-based therapy for MIBC and mBC in daily practice, in a large teaching hospital with a regional function for treatment of BCa in the Netherlands. These real-world outcomes were compared to data from RCTs and other retrospective cohorts (Table 4)^1,4,5,7,8,17,19,20,24,25^.

**Table 4:** Survival compared to literature on prospective clinical trials and retrospective observational studies.

Setting	Current study	Clinical Trial Setting	Observational
	*Median OS [95%CI]*	*Median OS*	*Median OS*
**MIBC or locally advanced disease**			
RC alone	47.4 months [28.9–65.8]	46 months^1^	54 months^19^
NAC + RC	35.3 months [5.6–64.9]*	77 months^1^	68 months^19^
IC + RC	19.5 months [0.0–49.1]	–	18–26 months^20^
Best Supportive Care	3.8 months [3.2–4.4]	–	5.3 months^17^
**Metastatic or inoperable disease**			
First-line chemotherapy (1L)	12.6 months [8.8–16.4]	14.0–15.2 months^4^	16.1 months^24^
		12.7–15.8 months^5^	
Recurrent disease after RC	7.5 months [0.0–15.6]	5.2 months^25^	-
Second-line chemotherapy (2L)	6.6 months [4.7–8.4]	5.9–7.0 months^7,8^	9.2 months^24^
Best Supportive Care (BSC)	2.0 months [0.9–3.2]	4.3–4.6 months^5,7^	-

*MIBC* muscle-invasive bladder cancer, *RC* radical cystectomy, *NAC* neoadjuvant chemotherapy, *IC* induction chemotherapy, *95% CI* 95% confidence interval.

*Patients with cT2N0M0 are not treated with NAC before RC in our hospital, and thus not present in this group.

The results of the present study, show a mOS of 47.4 months [95% CI 28.9–65.8], for patients undergoing RC alone. The prospective trial from Grossman et al. reported on a mOS of 46 months, in T2-4aN0M0 BCa patients receiving upfront RC versus 77 months for NAC before RC^1^. Similar results can be seen in stage II BCa patients from the ICOT-trial. Also, a retrospective study on 36.469 N0 MIBC patients, showed a mOS of 48 months after RC (with or without NAIC)^17^.

Patients treated with NAIC before RC do have poorer survival compared to literature. In node-negative MIBC patients receiving NAC before RC in the present study, survival was 35.3 months, [95% CI 5.6–64.9]), and thus a survival benefit similar to studies of Grossman et al. and the ICOT-trial was not seen^1,2^. This can be explained by the fact that cT2N0M0 patients are not present in this group. Also, an analyses of disease stage of the NAIC group shows a high disease stage (50% cT4). The overall survival of this group is not carried by cT2N0M0 patients, which are highly prevalent in the RC alone group, and as a result, overall survival is poorer.

The use of NAIC before RC is much lower, compared to RCTs. There is a reserved approach in the Netherlands using NAC in cT2N0M0 patients^26^. It is believed delayed cystectomy might compromise outcome in patients not sensitive to chemotherapy. Secondly, the presence of micro metastases is postulated to be lower in lower T-stage disease (cT2) compared to extensive tumours (cT3-4). Hermans et al. showed an increase in NAC in the Netherlands from 0.6% in 1995, to 21% in 2013^26^, similar to an analyses of the National Cancer Data Base registry in the United States^27^. When regarding > cT2N0M0 patients, 16% in the current study received NAIC before RC, or 37% of cT2-4N+ patients. Therefore, this study shows that use of NAIC in real-world patients remains low in the Netherlands.

Results show that many patients require dose reduction, and similarly early termination of chemotherapy cycles occurred in 21%, 42%, 38% and 50% of patients treated in NAIC, 1L, salvage after RC, and 2L setting, respectively. As a results, only 50%, 13%, 18% and 7% of patients in NAIC, 1L, salvage after RC, and 2L setting, could complete the full pre-planned chemotherapy treatment. Also, patients are often treated with carboplatin, which is considered inferior compared to cisplatin^28–30^. Especially in the palliative setting, the combination of these factors results in poor response on chemotherapy treatment. When guiding patients in deciding for NAIC before RC vs. RC alone or palliative chemotherapy vs. BSC, it is possible that the combination of patients’ difficulty in completing chemotherapy treatment, the highly prevalent complication rate and expected clinical response are the cause for renunciation of chemotherapy treatment during shared decision making.

Current study shows a lower OS compared to literature on clinical trials^4,5,30^ and retrospective^24^ studies in patients with mBC who received 1L chemotherapy (i.e. 12.6 months). However, when regarding only patients treated with GEM-CIS, median overall survival is 15.7 months, [95% CI 12.4–18.9], which is more similar.

In literature on RCTs, older and ‘unfit’ patients are underrepresented^14–16^. The question is whether treatment results from RCTs with strict in- and exclusion criteria are translatable to real-world patients, which do include these older and ‘unfit’ patients. In the entire MIBC and mBC cohort of this study, only 47% of patients were younger than 70 years of age. Elderly cancer patients are a heterogeneous group with respect to overall health status, due to differences in comorbidities, functional status, geriatric syndromes and socioeconomic aspects. Not all individuals over the age of 65 should be considered ‘elderly’^31^. In the Netherlands, it is expected that the age groups 65–79 and > 80 years will increase substantially. In 2040, 26% of the Dutch population is older than 65, with a third of that group older than 80 years of age (i.e. around 1.5 million people)^32^. When BCa is becoming a disease of the elderly, evidence is required in order to justify treatment strategies, to improve cancer care in these patients, and to adapt treatment to health status. This study shows that although treatment efficacy between RCTs and real-world patients is quite similar, there are large differences in baseline patient characteristics and treatment patterns. In this way, results from retrospective cohort can be used complementary to the results of RCTs. Multi-center, double-blinded RCTs, which aims to include a large study population, can be difficult to set up. When results from multi-center, large retrospective cohorts show similar oncological outcomes, perhaps these studies can provide evidence to justify treatment strategies more conveniently. Current study however, is limited by the small number of patients. More data is required to perform subanalyses to study treatment and oncological outcomes in elderly patients with poor performance score, and renal impairment.

Essential in retrospective studies is to include patients who did not receive treatment. In this study, 19% of patients diagnosed with MIBC did not receive curative treatment. These results vary with a study on the U.S. National Cancer Data Base, where 48% of patients with T2-T4aN0-3 MIBC received no curative therapy^33^. Similar to our study, 50% of patients were > 70 years of age. This difference could be the results of different health care system in the Netherlands and the United States. However, in metastatic setting, patients received chemotherapy treatment in 38% of cases, which is in line with another previously published study (42%)^34^. Patients who are disputable eligible for treatment can lower oncological outcomes of a treatment group when treated, or vice versa strengthen oncological outcomes when receiving BSC. Our study shows OS in patients receiving BSC in both the MIBC/locally advance arm, as well as the metastatic/inoperable disease arm to be lower compared to literature^5,7,17^. It is possible the ‘better’ patients in this group did receive a form of treatment, resulting in transition bias.

Better understanding of treatment outcomes for real-world patients, especially elderly and ‘unfit’ patients, can be achieved by standardizing patient outcome measures^35^, and measure these prospectively in registries. Standardized set of measured outcomes have been developed in the past, such as for both localized and advanced prostate cancer^36,37^. These sets encompass multiple treatment modalities, and can give results of larger population groups, nearly impossible to reach with RCTs. Especially with the emerging immunotherapeutic approach, with five checkpoint inhibitors gaining FDA approval for 2L therapy^38^, knowledge about outcomes in real-world settings is crucial for responsible application of these costly agents.

Strengths of this study are high resolution data, long observation and follow-up period and to the best of our knowledge one of the first descriptions of outcomes from routine clinical practice in the Netherlands. This study also has several limitations. Due to the retrospective nature of the study, selection bias may exist. However, the NCR database is expected to be a complete database. Secondly, the sample sizes are small, so limited statistical analyses were conducted and the setting of a single centre study precluded comparison between hospital sites.

## Conclusion

This retrospective study on chemo-based therapy for MIBC and mBC patients in the Netherlands, show that NAIC before RC is underutilized. Adversities during chemotherapy treatment are frequent, with many patients requiring dose reduction or early treatment termination, resulting in poor treatment response. Although, treatment efficacy between RCTs and real-world patients is quite similar, there are large differences in baseline characteristics and treatment patterns. Results from multi-center RCTs are often restricted for selected patients. Possibly, results from retrospective studies on real-world data can deliver missing evidence on efficacy of chemotherapy treatment on older and ‘unfit’ patients.

## Supplementary information

Supplementary file 1.
