# Supplementary material for: Treatment patterns and clinical outcomes of chemotherapy treatment in patients with muscle-invasive or metastatic bladder cancer in the Netherlands
Source: Sci Rep. 2020 Sep 25;10:15822. doi: 10.1038/s41598-020-72820-y (PMC7519076; doi:10.1038/s41598-020-72820-y)
Supplement: Supplementary file 1 [file 41598_2020_72820_MOESM1_ESM.docx]

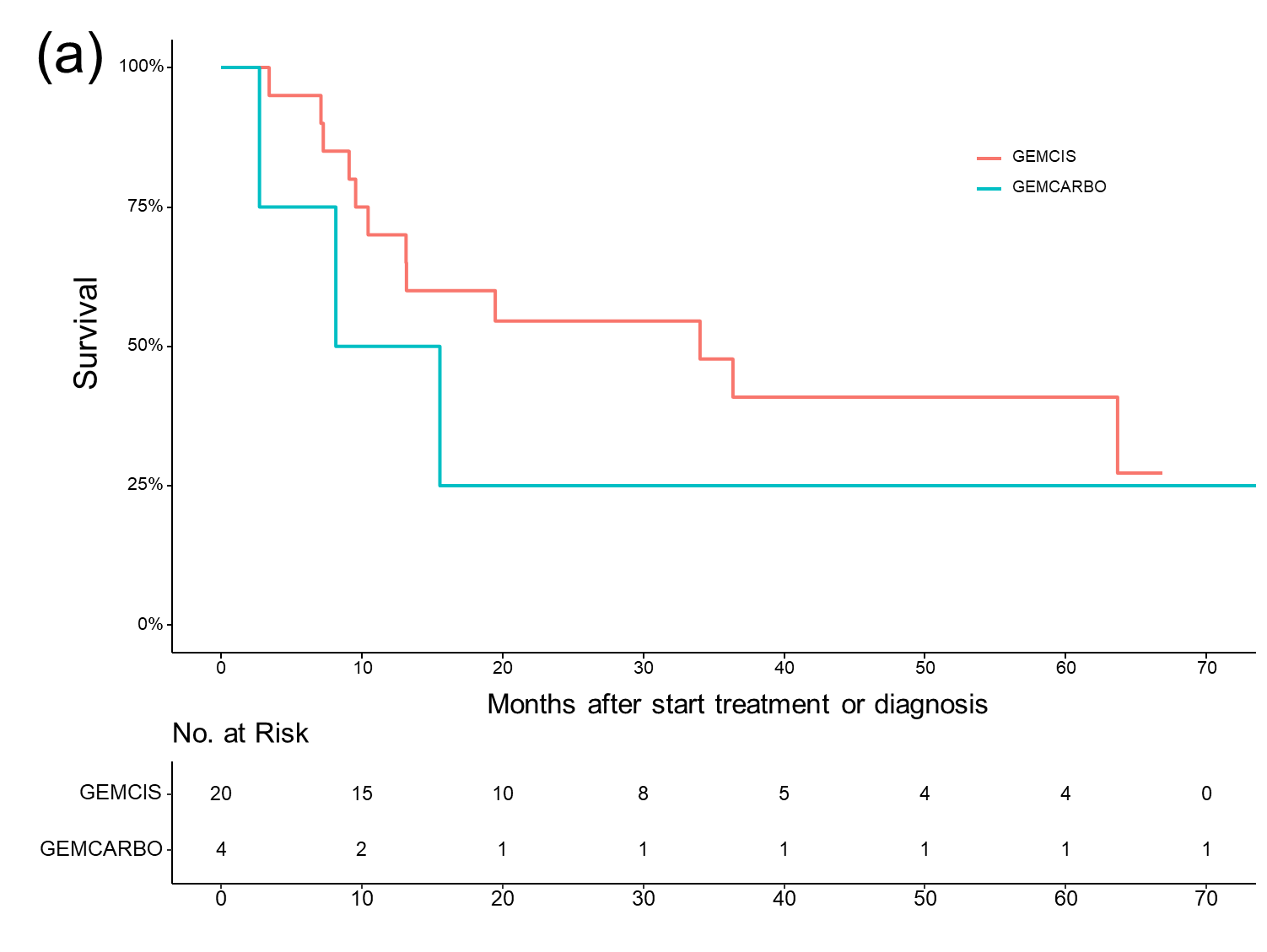

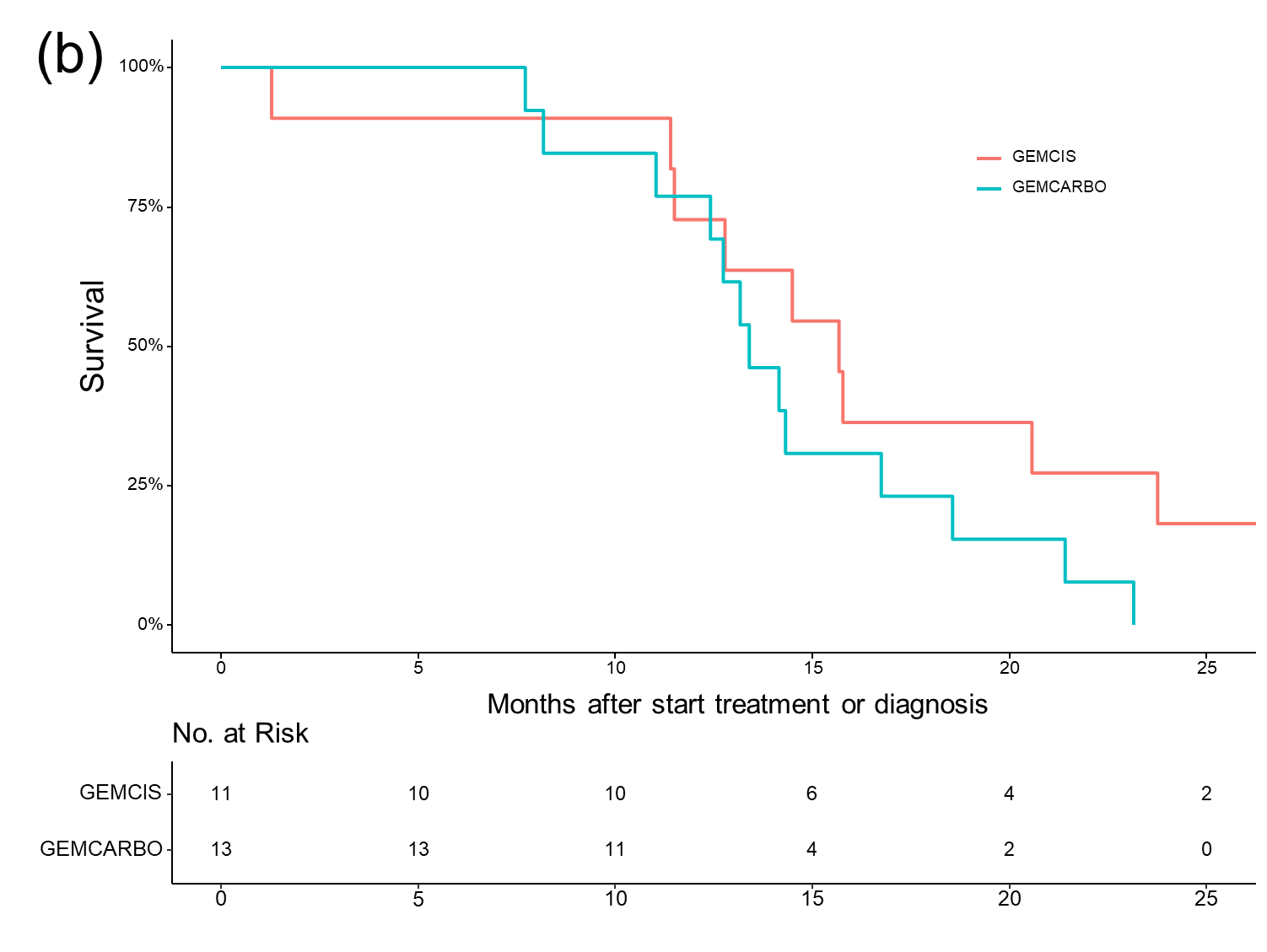


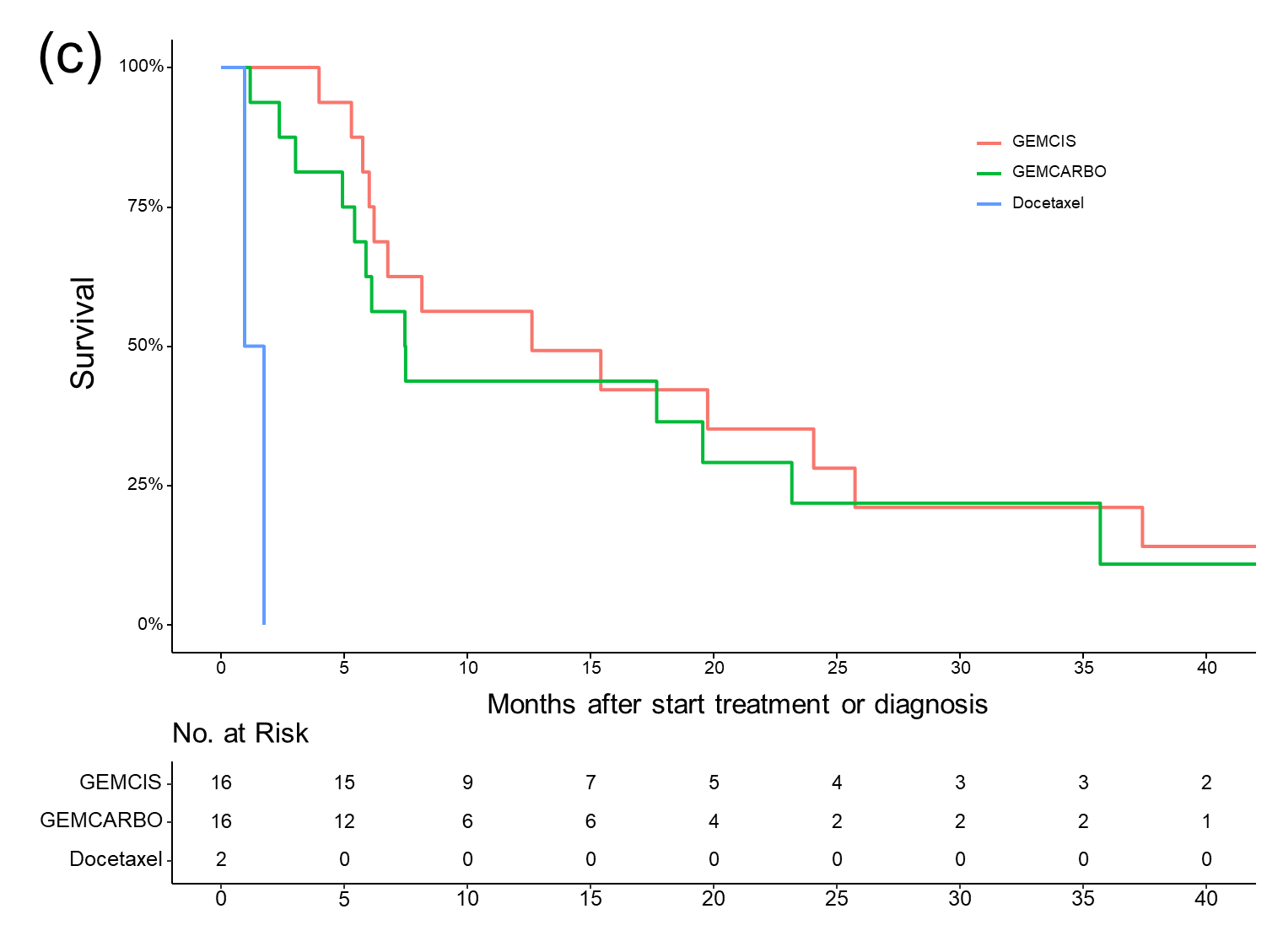

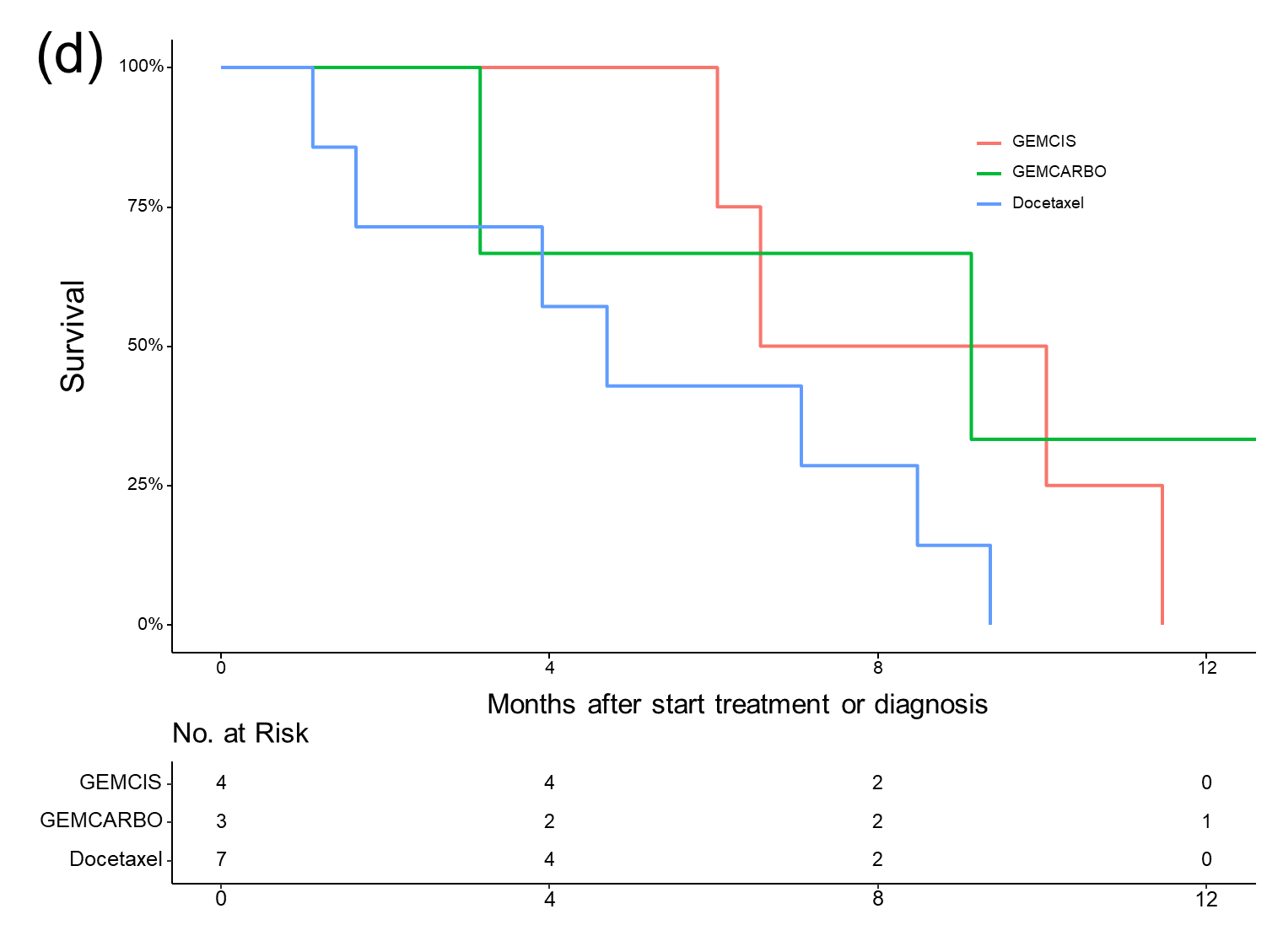


Supplementary Figure 3a-d. Kaplan-Meier curves showing overall survival for (a) Neoadjuvant/Induction chemotherapy (NAIC), (b) First-line (1L) chemotherapy for metastatic disease, (c) Salvage chemotherapy after recurrent disease after radical cystectomy and (d) Second-line (2L) chemotherapy, stratified for Gemcitabine + Cisplatin (GEMCIS), Gemcitabine + Carboplatin (GEMCARBO), or Docetaxel.
